# A systematic review on the latest developments in testosterone therapy: Innovations, advances, and paradigm shifts

**DOI:** 10.1080/2090598X.2021.1959260

**Published:** 2021-08-08

**Authors:** Raed M. Al-Zoubi, Aksam A. Yassin, Mustafa Alwani, Ahmad Al-Qudimat, Omar M. Aboumarzouk, Ahmad Zarour, Abdulla Al Ansari

**Affiliations:** a Surgical Research Section, Department of Surgery, Hamad Medical Corporation, Doha, Qatar; b Department of Chemistry, Jordan University of Science and Technology, Irbid, Jordan; c Department of Surgery, Division of Urology/Andrology, Hamad Medical Corporation, Doha, Qatar; d Center of Medicine and Health Sciences, Dresden International University, Dresden, Germany; e School of Medicine, Jordan University of Science and Technology, Irbid, Jordan

**Keywords:** Hypogonadism, testosterone therapy, ageing male, sexual health, obesity, prostate health, diabetes

## Abstract

**Objectives:**

To review the latest innovations and advances in testosterone treatments including their advantages and disadvantages and to address important issues in testosterone therapy (TTh).

**Methods:**

This review was conducted according to the Preferred Reporting Items for Systemic Reviews and Meta-analyses guidelines. The PubMed, MEDLINE, Scopus and Cochrane databases were searched using specifically related key words. The identiﬁed studies were screened for inclusion criteria that included studies discussing one of the four objectives of the systematic review: 1) cut–oﬀ references, 2) prevention/remission of type II diabetes mellitus (T2DM), 3) duration of treatment, and 4) prostate, lower urinary tract symptoms, prostate health, or cancer. The search was limited to the past 15 years. Any studies were not written in English were excluded.

**Results:**

The initial literature search retrieved 393 studies. After screening four studies were removed due to duplication, 360 studies were further excluded after reviewing the title, abstract or the whole manuscript due to diﬀerent exclusion criteria or being not focussed on the objective. Finally, 29 studies were included in the review. One study discussed the cut–oﬀ value, four studies discussed the eﬀect of testosterone replacement therapy (TRT) on the control of T2DM, four studies on duration of TRT, and 20 studies discussed the eﬀects of TRT on the prostate

**Conclusions:**

Numerous studies have demonstrated the beneﬁts of TTh in overtly hypogonadal men. There are several possible administration routes for testosterone treatment. Each approach has advantages and disadvantages, and the choice of the method of TRT will often be determined by patient preference or co-medication (no intramuscular injections in patients under coumarin or similar anticoagulants). Although new developments are promising, it seems that among the available treatments, only transdermal gel delivery and long-acting injectable testosterone undecanoate provide pharmacokinetic behaviour that gives a steady state level within a physiological range.

## Introduction

During the last two decades, there has been a revolution in therapeutic treatment options to provide healthcare providers and their hypogonadal patients the best treatment option when aiming to restore serum testosterone to physiological concentrations. Different therapeutic options have been reported from implanted testosterone pellets to injectable testosterone esters, short and long acting and then to oral methyltestosterone. The first-generation oral testosterone undecanoate (TU) product then to scrotal and non-scrotal testosterone patches and then to topical testosterone gels [1]. Other recent testosterone replacement therapy (TRT) innovations include a long-acting TU injection (intramuscular [IM]) and a short-acting testosterone enanthate injection (hypodermal) [2] and a nasal testosterone gel. The oral soft gel formulation of TU is the latest innovation in testosterone therapy (TTh) and recent reports of phase III clinical studies have shown that this new formulation is safe and effective as a therapeutic in the treatment of men with hypogonadism [3]. Two synthetic paths were used to make the oral testosterone: the alkylation of the C-17 position using chemical modification of testosterone, to make the tertiary alcohol derivative, 17 α-methyl-testosterone (methyltestosterone) that is found to resist hepatic metabolism and cause potentially serious liver toxicity or by the esterification reaction of testosterone using fatty acid to make the ester-linkage testosterone derivative as IM injections (testosterone enanthate, cypionate, and TU) and for oral use (TU only). Although TU has been found to be absorbed via the intestinal lymphatic system and has been widely available and used outside the USA, due to its pharmacokinetic profile it has never been approved for use in the USA [2].

For TTh, gels and long-acting TU 1000 mg can both help to bring the testosterone level to a steady state physiological concentration, with the long-acting IM injections (TU 1000 mg for quarterly IM injections, available since November 2004) reaching higher physiological levels, which results in more profound clinical effects and preferable benefits on different organ systems.

The innovations and advances made in TTh in the last two decades can be categorised in to four main areas: 1) Cut-off references; 2) Prevention/remission of type II diabetes mellitus (T2DM); 3) Duration of treatment (or how long should last TTh?); and 4) Prostate, LUTS, prostate health, cancer (paradigm shift). In addition, advances have been made in enhancing the role of testosterone as a metabolic hormone with favourable effects on 1) Sexual function; 2) Obesity; 3) Muscles vs fats; 4) Bone health; 5) Blood formulation (anaemia); 6) cardiovascular effects and blood pressure; 7) Renal function; 8) Liver function and steatosis; and 9). Depression.

The aim of the present review was to consolidate the recent data on the four main innovations in TTh.

## Methods

This review was conducted according to the Preferred Reporting Items for Systemic Reviews and Meta-analyses (PRISMA) guidelines. The PubMed, MEDLINE, Scopus, and Cochrane databases were searched using the following keywords: (‘testosterone’ or ‘testosterone replacement’) AND ‘diabetes’ OR ‘Prostate’, ‘prostate cancer’ OR ‘interrupted’ OR ‘interruption’ OR ‘LUTS’ OR ‘cut-off’ OR ‘reference range’. Studies recruited were screened for inclusion criteria, which included studies discussing one of the four objectives of the systematic review:

1. Cut off references,
2. Prevention/remission of T2DM
3. Duration of treatment
4. Prostate, LUTS, prostate health or cancer.

**Figure 1.**
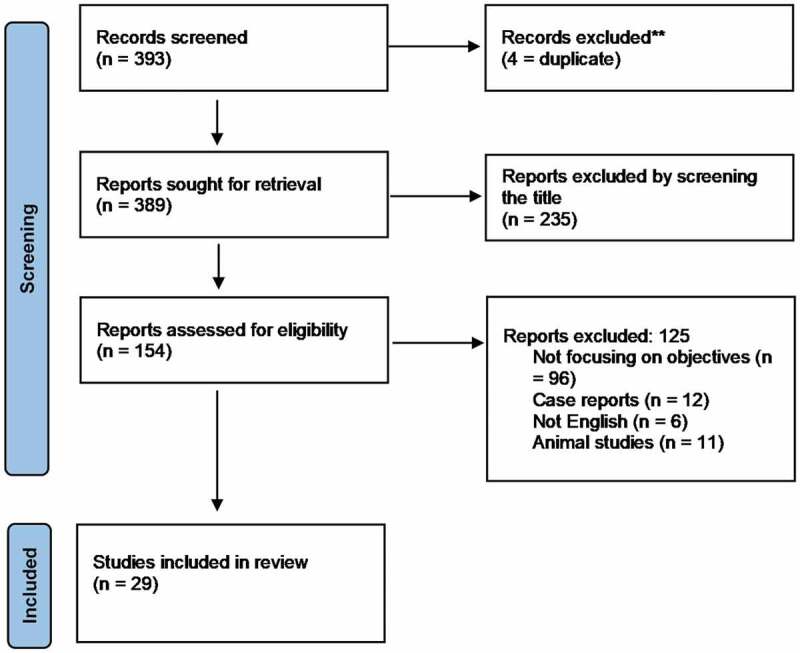
PRISMA flow diagram

The search was limited to the past 15 years. Animal studies, case reports and studies not written in English were excluded. Screening was done by two researchers individually. In case of discrepancy between them, a senior researcher would review it (Figure 1).

## Results

The initial literature search retrieved 393 studies. After screening four studies were removed due to duplication, 360 studies were further excluded after reviewing the title, abstract or the whole manuscript due to different exclusion criteria or being not focussed on the objective. A total of 29 studies were ultimately included in the review. One study discussed the cut-off value, four studies discussed the effect of TRT on control of T2DM, four studies on duration and interruption of TRT, and 20 studies discussed effect of TRT on the prostate.

## Areas of concentration of four major innovations

### Cut-off or threshold (normal testosterone range)

Reference ranges are essential for partitioning testosterone levels into low or normal and making the diagnosis of androgen deficiency. Bhasin et al. [4] established reference ranges for total testosterone (TT) and free testosterone (FT) in a community-based sample of men. These reference ranges generated in a community-based sample of men provide a rational basis for categorising testosterone levels as low or normal. Men with low TT or FT by these criteria have a higher prevalence of physical dysfunction, sexual dysfunction, and DM. These reference limits are 12.1–33 nmol/L for TT and 70–141 pg/mL for FT [4].

### Prevention/remission of T2DM

Type II DM is often associated with obesity and subnormal serum testosterone levels. Until recently, there was no indication that men with type I DM had subnormal serum testosterone levels. Studies indicated, as a rule in aged men and men with obesity, that ~10% of men with type I DM have hypogonadism.

In 2019, Yassin et al. [5] reported on long-term TRT in men with hypogonadism for an 8-year period and found that it completely prevented the progression of pre-DM to overt T2DM in men with hypogonadism and pre-DM. TRT also resulted in a marked reduction of cardiovascular disease (CVD) risk by reducing body weight, waist circumference, and glycaemia and improving dyslipidaemia.

In 2018, Morgunov et al. [6] studied hypogonadism and its treatment following ischaemic stroke in men with T2DM. The authors found that TRT was a useful clinical tool to manage ischaemic events in this subset of patients, whilst having a potentially positive effect in their mobility and the overall quality of life. This study recommended that with careful monitoring, testosterone-deficient patients with T2DM and cardiovascular risk may benefit from TRT.

Other observational studies of pooled analyses in obese hypogonadal men with T2DM found that TRT significantly reduced fasting blood glucose and haemoglobin subunit alpha 1 c (HbA1c) levels, reduced total cholesterol, low-density lipoprotein cholesterol, triglycerides, levels of inflammatory markers and suggesting reduction in the inflammation response and increased high-density lipoprotein cholesterol levels and improved systolic and diastolic blood pressure. TRT of obese, diabetic men improves glycaemic control and lipid profiles and may prove useful in reducing the risk of CVD [7]. All these data [5–7] were recently validated in a placebo-controlled study over 2 years published in January 2021 [8].

### Duration of treatment (or how long should we treat with testosterone?)

Four papers discussed this key question on ‘How long should we treat with testosterone?’.

Our observation indicates that testosterone administration improves body weight and metabolic factors in men with hypogonadism, but withdrawal of testosterone reverses these beneficial effects, which appear again when TTh is resumed [9,10].

A total of 262 hypogonadal patients (mean age 59.5 years) received TU in 12-week intervals for a maximum of 11 years. Of these men, 147 had their TRT interrupted for a mean of 16.9 months and resumed thereafter (Group A). The remaining 115 patients were treated continuously (Group B). Prostate volume, PSA, residual voiding volume, bladder wall thickness, C-reactive protein (CRP), Aging Males’ Symptoms (AMS) scale, International Index of Erectile Function-erectile function (IIEF-EF) and IPSS were measured over the study period with anthropometric parameters of obesity, including weight, body mass index and waist circumference. Prior to interruption, TRT resulted in improvements in residual voiding volume, bladder wall thickness, CRP, AMS, IIEF-EF, IPSS and obesity parameters, while PSA and prostate volume increased. TRT interruption reduced TT to hypogonadal levels in Group A and resulted in worsening of obesity parameters, AMS, IPSS, residual voiding volume and bladder wall thickness, IIEF-EF, and PSA, while CRP and prostate volume were unchanged until treatment resumed whereby these effects were reversed. These observations indicate that testosterone administration improves body weight and metabolic factors in men with hypogonadism, but withdrawal of testosterone reverses these beneficial effects, which reappear when TRT is resumed. TRT interruption results in worsening of symptoms, thus hypogonadism may require lifelong TRT [11].

### Prostate health and cancer (paradigm shift)

For a long time, it was believed that higher testosterone concentrations increased the risk of prostate cancer or caused rapid cancer growth, while low testosterone concentrations would have a protective outcome. PSA is often used as a marker of prostate health, and several studies have investigated this association and at how TRT affects PSA. Most of these studies found that increased testosterone even over the long term does not affect PSA or its effect to be negligible [12–15].

A prostate saturation hypothesis by Morgentaler and Traish [16] may explain these results, that when androgen receptors on the prostate become ‘saturated’, the prostate becomes insensitive to further serum testosterone increases, such as with TRT. A placebo-controlled trial study by Marks et al. [17] found an increase in serum testosterone and dihydrotestosterone when men were on IM TRT for 6 months; however, when biopsied before and after TRT, there was no change in androgen levels within the prostate tissue. PSA is not the perfect marker for prostate cancer; so, several studies have tried to clarify if TRT increases the occurrence of prostate cancer. A plethora studies have not demonstrated testosterone concentrations to be higher in men with prostate cancer compared to those without cancer. Moreover, reports have shown that hypogonadal men with normal PSA levels do not have lower cancer rates than the general healthy population [18].

Haider et al. [19] reported the observations of three registries on the incidence of prostate cancer in 1023 hypogonadal men receiving TRT followed-up for 5 years. The authors suggest that TRT does not increase the risk of prostate cancer. Yassin et al. [20] showed that TRT does not increase the risk for prostate cancer when compared to healthy men and recommended further investigations to truly understand the complex relationship between prostate cancer and testosterone. Other studies investigated the effects of TRT in patients who had prostate cancer especially those who were diagnosed with prostate cancer but untreated. A prospective data study on 553 patients who underwent prostate biopsy to investigate the role of TRT in prostate safety and cancer progression. The authors found that the incidence of positive prostate biopsies was lowest in hypogonadal men receiving TRT and with lower prostate cancer severity in terms of staging. These results suggest that TRT might have a protective effect against high-grade prostate cancer [21,22].

In fact, several authors found no recurrences while on TRT, suggesting that TRT can be safe regardless of risk [20,21,23,24].

On the other hand, many studies discussed and examined the influence of testosterone administration on symptom scores of LUTS and on BPH. In a review by Yassin et al. [25] in 2006, the authors referred to some studies investigating the effects of normalising testosterone levels in elderly men and found that it had a positive effect on variables of the metabolic syndrome and, simultaneously, on scores of the IPSS (which is worthy of further investigation in randomised, controlled and sufficiently powered clinical trials). Other studies followed, stating the relationship between erectile dysfunction and LUTS and TTh alone or in combination with α-blockers or phosphodiesterase type 5 inhibitors (PDE-5i) can improve both erectile dysfunction and LUTS [26]. Studies on interruption of TTh and resumption clearly showed the benefits on LUTS and voiding symptoms, where authors stated that interruption reduced TT to hypogonadal levels resulting in worsening of obesity parameters, AMS, IPSS, residual voiding volume and bladder wall thickness, IIEF-EF, and PSA while CRP and prostate volume were unchanged until treatment resumed whereby these effects were reversed. TTh interruption results in worsening of symptoms. Hypogonadism may require lifelong TTh [9–11].

The traditional assumption that the prostate is an exquisitely sensitive organ to androgen action still holds true, but there are several new insights:

- The saturation model: with lower-than-normal circulating levels of testosterone, all androgen receptors are saturated and a further increase in circulating levels of testosterone has no effect on the prostate.
- This has relevance for prostate disease (prostate cancer and BPH) usually occurring at an age when circulating levels of testosterone are declining. These diseases cannot be attributed to an excess of testosterone.
- It is customary now not to attribute the bother elderly men experience with micturition to the prostate only, but to subsume this under pathology of the lower urinary tract. Surprisingly, these structures have androgen receptors and for their functioning they depend on nitric oxide (NO) for the relaxation of smooth muscle structures, having this in common with the biological substrate of erectile function. This explains why PDE-5i benefit both erectile function and LUTS [26–28].

Testosterone augments the action of NO and therefore testosterone might be helpful in men with LUTS who are testosterone deficient. It becomes apparent that testosterone is not only significant for the formation of male urogenital anatomical structure prenatally, their growth and functioning at the time of puberty but that these structures also need testosterone for maintaining their normal functioning. TTH might improve irritative and obstructive symptoms independent from prostate size [29].

## Conclusion

Testosterone deficiency is associated with adverse effects on body composition, bone density, sexual function, and mood and may also increase insulin resistance, fatty liver, and cardiovascular risk factors deterioration. Numerous studies have shown the benefits of TTh overtly in hypogonadal men. There are several possible administration routes for testosterone treatment. Each approach has advantages and disadvantages, and the choice of the method of replacement will often be determined by patient preference or co-medication (e.g. no IM injections in patient under coumarin or similar anticoagulants). Although new developments are promising, it seems that, among the available treatments, only transdermal gels delivery and long-acting injectable TU have provided pharmacokinetic behaviour that gives a steady state level within the physiological range.

Hypogonadism or testosterone deficiency is a significant medical concern and a systemic disease that can impact on multiple organ systems causing morbidities and negatively affect overall quality of life with associated increases in the incidence of sexual dysfunction, metabolic syndrome, DM, obesity, depression, and others. Due to the above-mentioned advantages, TTh has clearly gained popularity among hypogonadal males as a safe and beneficial treatment, with increasing evidence of favourable effects on multiple organ systems. Combined therapy with testosterone and other treatments, such as PDE-5i, is advantageous in some cases and is valuable for patients with hypogonadism who failed PDE-5i therapy alone.

## Abbreviations

AMS: Aging Males’ Symptoms; CRP: C-reactive protein; CVD: cardiovascular disease; (T2)DM: (type II) diabetes mellitus; FT: free testosterone; IIEF-EF: International Index of Erectile Function-erectile function; NO: nitric oxide; PDE-5i: phosphodiesterase type 5 inhibitors; PRISMA: Preferred Reporting Items for Systemic Reviews and Meta-analyses; TTh: testosterone therapy; TRT: testosterone replacement therapy; TT: total testosterone; TU: testosterone undecanoate

## References

[1] Saad F, Gooren LJ, Haider A, et al. An exploratory study of the effects of 12-month administration of the novel long-acting testosterone undecanoate on measures of sexual function and the metabolic syndrome. Arch Androl. 2007 Nov-Dec;53(6):353–357. 18357966 10.1080/01485010701730880

[2] Yassin AA, Haffejee M. Testosterone depot injection in male hypogonadism: a critical appraisal. Clin Interventions Aging. 2007;2(4):577–590. PMC2686335 18225458

[3] Swerdloff RS, Dudley RE. A new oral testosterone undecanoate therapy comes of age for the treatment of hypogonadal men. Ther Adv Urol. 2020;12:1–16. 10.1177/1756287220937232 PMC7328356 32655691

[4] Bhasin S, Pencina M, Jasuja GK, et al. Reference ranges for testosterone in men generated using liquid chromatography tandem mass spectrometry in a community-based sample of healthy nonobese young men in the Framingham heart study and applied to three geographically distinct cohorts. J Clin Endocrinol Metab. 2011 Aug;96(8):2430–2439. Epub 2011 Jun 22. . 21697255 10.1210/jc.2010-3012 PMC3146796

[5] Yassin A, Haider A, Haider K, et al. TTh in men with hypogonadism prevents progression from prediabetes to type 2 diabetes: eight-year data from a registry study. Cardiovasc Metab Risk. Published. 1 June 2019. DOI:10.2337/dc18-2388. 30862651

[6] Morgunov L, Denisova I, Rozhkova T, et al. Hypogonadism and its treatment following ischaemic stroke in men with type 2 diabetes mellitus. Skvortsova (2018): hypogonadism and its treatment following ischaemic stroke in men with type 2 diabetes mellitus. Aging Male. DOI:10.1080/13685538.2018.1487932 30064273

[7] Haider A, Yassin A, Doros G, et al. Effects of long-term tth on patients with “diabesity”: results of observational studies of pooled analyses in obese hypogonadal men with type 2 diabetes. Int J Endocrinol. 2014;2014:683515. 24738000 10.1155/2014/683515 PMC3967627

[8] Wittert G, Bracken K, Robledo KP, et al. Testosterone treatment to prevent or revert type 2 diabetes in men enrolled in a lifestyle programme (T4DM): a randomised, double-blind, placebo-controlled, 2-year, phase 3b trial. Lancet Diabetes Endocrinol. 2021;9(1):32–45. . 33338415 10.1016/S2213-8587(20)30367-3

[9] Yassin A, Nettleship JE, Talib RA, et al. Effects of testosterone replacement therapy withdrawal and re-treatment in hypogonadal elderly men upon obesity, voiding function and prostate safety parameters. Aging Male. 2016;19(1):64–69. Epub 2016 Jan 8. . 26742589 10.3109/13685538.2015.1126573

[10] Saad F, Yassin A, Almehmadi Y, et al. Effects of long-term testosterone replacement therapy, with a temporary intermission, on glycemic control of nine hypogonadal men with type 1 diabetes mellitus – a series of case reports. Aging Male. 2015;18(3):164–168. 26075537 10.3109/13685538.2015.1034687

[11] Yassin A, Almehmadi Y, Saad F, et al. Effects of intermission and resumption of long-term testosterone replacement therapy on body weight and metabolic parameters in hypogonadal in middle-aged and elderly men. Clin Endocrinol (Oxf). 2016 Jan;84(1):107–114. . 26331709 10.1111/cen.12936

[12] Raynaud JP, Gardette J, Rollet J, et al. Prostate-specific antigen (PSA) concentrations in hypogonadal men during 6 years of transdermal testosterone treatment. BJU Int. 2013;111(6):880–890. PSA did not change nor did the incidence of PCa increase significantly while on TRT. It has never been shown that TRT leads to enhanced prostate growth. . 23294726 10.1111/j.1464-410X.2012.11514.x

[13] Khera M, Bhattacharya RK, Blick G, et al. Changes in prostate specific antigen in hypogonadal men after 12 months of Testosteronetherapy: support for the prostate saturation theory. J Urol. 2011;186(3):1005–1011. 21788049 10.1016/j.juro.2011.04.065

[14] Ho CC, Tong SF, Low WY, et al. A randomized, double-blind, placebo-controlled trial on the effect of long-acting testosterone treatment as assessed by the aging male symptoms scale. BJU Int. 2012;110(2):260–265. 22093057 10.1111/j.1464-410X.2011.10755.x

[15] Kaufman JM, Miller MG, Garwin JL, et al. Efficacy and safety study of 1.62% testosterone gel for the treatment of hypogonadal men. J Sex Med. 2011;8(7):2079–2089. 21492400 10.1111/j.1743-6109.2011.02265.x

[16] Morgentaler A, Traish AM. Shifting the paradigm of testosterone and prostate cancer: the saturation model and the limits of androgen-dependent growth. Eur Urol. 2009;55(2):310–320. 18838208 10.1016/j.eururo.2008.09.024

[17] Marks LS, Mazer NA, Mostaghel E, et al. Effect of testosterone therapy on prostate tissue in men with late-onset hypogonadism: a randomized controlled trial. JAMA. 2006;296(19):2351–2361. 17105798 10.1001/jama.296.19.2351

[18] Morgentaler A, Bruning C, DeWolf W. Occult prostate cancer in men with low serum testosterone levels. Jama. 1996;276:1904–1906. 8968017

[19] Haider A, Zitzmann M, Doros G, et al. Incidence of prostate cancer in hypogonadal men receiving testosterone therapy: observations from 5-year median followup of 3 registries. J Urol. 2015 Jan;193(1):80–86. Epub 2014 Jun 26. PMID: 24980615. . 24980615 10.1016/j.juro.2014.06.071

[20] Yassin A, AlRumaihi K, Alzubaidi R, et al. Testosterone, testosterone therapy and prostate cancer. Aging Male. 2018;22(4):219–227. 10.1080/13685538.2018.1524456 30614347

[21] Yassin A, Salman M, Talib R, et al. Is there a protective role of testosterone against high-grade prostate cancer? Incidence and severity of prostate cancer in 553 patients who underwent prostate biopsy: a prospective data register. Aging Male. 2017;20(2):125–133. 28282997 10.1080/13685538.2017.1298584

[22] Pastuszak AW, Rodriguez KM, Nguyen TM, et al. Testosterone therapy and prostate cancer. Transl Androl Urol. 2016;5(6):909–920. 28078223 10.21037/tau.2016.08.17 PMC5182214

[23] Morgentaler A, Rhoden EL. Prevalence of prostate cancer among hypogonadal men with prostate-specific antigen levels of 4.0 ng/mL or less. Urology. 2006 Dec;68(6):1263–1267. PMID: 17169647. . 17169647 10.1016/j.urology.2006.08.1058

[24] Morgentaler A, Caliber M. Safety of testosterone therapy in men with prostate cancer. Expert Opin Drug Saf. 2019 Nov;18(11):1065–1076. 31495240 10.1080/14740338.2019.1666103

[25] Yassin A, El-Sakka AI, Saad F, et al. Lower urinary-tract symptoms and testosterone in elderly men. World J Urol. 2008 Aug;26(4):359–364. Epub 2008 Jul 2. . 18594831 10.1007/s00345-008-0284-x PMC2517082

[26] Yassin A, Saad F, Hoesl CE, et al. Alpha-adrenoceptors are a common denominator in the pathophysiology of erectile function and BPH/LUTS – implications for clinical practice. Andrologia. 2006;38(38):1–12. 16420236 10.1111/j.1439-0272.2006.00709.x

[27] Vignozzi L 1, Morelli A, Sarchielli E, et al. Testosterone protects from metabolic syndrome-associated prostate inflammation: an experimental study in rabbit. J Endocrinol. 2012 Jan;212(1):71–84. Epub 2011 Oct 18. . 22010203 10.1530/JOE-11-0289

[28] Saad F, Yassin AA, Haider A, et al. Effects of testosterone on the lower urinary tract go beyond the prostate: new insights, new treatment options. Arab J Urol. 2011 Jun;9(2):147–152. Epub 2011 Sep 9. . 26579287 10.1016/j.aju.2011.06.003 PMC4150581

[29] Yassin A, Alrumaihi K, Talib R, et al. 186 Voiding function improves under long-term treatment with testosterone undecanoate injections (TU) in hypogonadal men for up to 14 years independent of prostate size. Eur Urol Suppl. 2019;18(1):e253.

